# Association Between Peripheral Adiponectin and Lipids Levels and the Therapeutic Response to Donepezil Treatment in Han Chinese Patients With Alzheimer’s Disease

**DOI:** 10.3389/fnagi.2020.532386

**Published:** 2020-09-11

**Authors:** Lili Wan, Jin Lu, Jinlu Huang, Yan Huo, Shan Jiang, Cheng Guo

**Affiliations:** ^1^Department of Pharmacy, Shanghai Jiao Tong University Affiliated Sixth People’s Hospital, Shanghai, China; ^2^Pamela Sklar Division of Psychiatric Genomics, Department of Genetics and Genomic Sciences, Icahn School of Medicine at Mount Sinai, New York, NY, United States; ^3^Icahn Institute for Data Science and Genomic Technology, Department of Genetics and Genomic Sciences, Icahn School of Medicine at Mount Sinai, New York, NY, United States; ^4^Shanghai Jiao Tong University School of Medicine, Shanghai, China

**Keywords:** Alzheimer’s disease, donepezil, adiponectin, methyl stearate, glycerol-3-phosphate, adipogenic differentiation

## Abstract

Acetylcholinesterase inhibitors (AChEIs) including donepezil (DNP) are considered to be the most promising therapeutic possibilities of Alzheimer’s disease (AD). The response to DNP in AD patients varies and it is valuable to identify the potential markers that can predict the efficacy. Moreover, DNP has been found to affect bone function, but the exact mechanism is still unclear. Lipids and adipokine may link to AD and DNP directly or indirectly and might be potential biomarkers or therapeutic drug targets. The goal of this study was to investigate the relationships among adiponectin (APN), lipids levels, and the response to DNP, and to identify whether the effect of DNP in AD treatment is related to its effect on the level of APN in systemic circulation. The study recruited 85 AD patients with DNP treatment, of whom 47 were DNP responders and 38 were DNP nonresponders. The Mini-Mental State Examination was performed to evaluate the memory impairment. Plasma APN was measured with ELISA. The genotypes of single nucleotide polymorphisms rs1501299 and rs22417661 in APN for each patient were identified. Plasma lipids were quantified with gas chromatography coupled with mass spectrometry. Correlations among APN, lipid metabolomics, and DNP responded were evaluated. APN was significantly decreased in DNP responders. Methyl stearate and glycerol-3-phosphate, used for characterizing adipogenic differentiation, were significantly decreased in DNP responders compared to DNP nonresponders. APN and small-molecule lipids can be used as potential biomarkers to evaluate the efficacy of DNP. The results of metabolomics indicated that there was no change in the metabolic pathway of fatty acid metabolism and glucose metabolism in DNP responders, suggesting that APN-related biological function did not decrease in DNP responders. Our result suggests that more attention should be pay to the sources and biological functions of APN in AD with DNP treatment.

## Introduction

Adiponectin (APN) is one of the most abundant adipokines, exerting protective effects such as insulin-sensitizing, antiatherogenic, and anti-inflammatory properties (Juhl and Beck-Sickinger, 2012). Many epidemiological studies have demonstrated that serum levels of APN were negatively correlated with body mass index (BMI), type 2 diabetes (T2D) and cardiovascular diseases (Caselli et al., 2014; Matsuda and Shimomura, 2014; Choi et al., 2015; Lindberg et al., 2015; Lubkowska et al., 2015; Han et al., 2017). Paradoxically, recent epidemiological studies reported that APN levels were increased in patients with chronic heart failure (Bozic et al., 2010; Celik and Yaman, 2010), anorexia nervosa (Baranowska-Bik et al., 2017; Legroux-Gérot et al., 2019; Tyszkiewicz-Nwafor et al., 2019), osteoporosis (Mpalaris et al., 2016; Tanna et al., 2017) and Alzheimer’s disease (AD; Khemka et al., 2014; García-Casares et al., 2016; Waragai et al., 2016; Wennberg et al., 2016). This is called the APN paradox, the mechanism of which is poorly understood. Better understanding of the APN paradox in AD might be critical from both mechanistic and therapeutic perspectives. Better understanding of the APN paradox, so unique a phenomenon of APN action in AD, can help us to find a novel therapeutic strategy that is distinct from previous therapy for AD and find a useful biomarker for evaluating AD progression. APN is primarily synthesized by adipocytes and plays a central role in lipid metabolism. To gain insights into the biology that regulates APN levels, an exome array meta-analysis found the candidate genes include RKAR2A, PTH1R, and HDAC9, which researchers have suggested play roles in adipocyte differentiation or the function of bone marrow adipose tissue (MAT; Spracklen et al., 2019). The coexistence of adipocytes, osteoblasts, and osteocytes in the bone marrow provides the possibility that adipocytes regulate bone homeostasis through paracrine and endocrine mechanisms, APN can be secreted from MAT and further regulate other adipose depots and whole body homeostasis. APN from marrow adipocytes plays a substantial contribution to circulating APN levels (de la Monte and Wands, 2006; Scheller et al., 2016). Growing evidence points to the close association between APN levels and bone-fat metabolism (Yang et al., 2019). Low bone-mineral density (BMD), osteoporosis, and increased fracture risk are significant clinical comorbidities in AD that may also be linked to bone marrow. It is intriguing to speculate that the fluctuation of APN from MAT might act as a driving force in the pathogenesis of AD, and APN might be the therapeutic drug target of AD. Elderly patients with AD who were at risk of developing osteoporosis may potentially benefit from therapy with acetylcholinesterase inhibitors (AChEIs; Tamimi et al., 2012). Whether APN can act as AChEIs or a potential drug target in AD treatment is not yet clear.

Donepezil (DNP), a cerebral selective acetylcholinesterase inhibitor, is the most widely administered medication for AD (Birks and Craig, 2006; Arvanitakis et al., 2019). However, not all patients respond to DNP therapy. The percentage of improved patients is about 50%. The clinical features of responders or nonresponders to long-term DNP treatment remained unclear (Giacobini, 2000). Furthermore, not all patients respond to DNP, and the prediction of treatment efficacy prior to the initiation of treatment is difficult. DNP acts primarily on the cholinergic system as symptomatic treatment, but it also has potential for attenuating pathological changes and may reduce the rate of progression of AD. Previous research has indicated that DNP could increase serum APN levels (Pákáski et al., 2014). The animal study showed that DNP may have other beneficial effects on body tissues favoring bone mass (Eimar et al., 2016). Administration of AChEIs is associated with clinically reduction in fracture risk in dementia patients (Tamimi et al., 2018; Won et al., 2020). APN from marrow adipocytes plays a role in the manipulation of the fate of mesenchymal stem cells as well as the osteoblastic activity during bone remodeling *via* direct and indirect effects. However, it is not clear whether APN has any effect on bone homeostasis. Moreover, whether the APN level reflects the underlying responsiveness to DNP in AD is unclear.

In the present study, we investigated the level of APN in DNP responders and nonresponders. We found lower APN levels in DNP responders than nonresponders in AD patients. To better understand the possible association between treatment response and APN levels, we performed a study using gas chromatography/mass spectrometry (GC-MS) for comprehensive metabolomic analysis. Metabolomics can provide a systematic insight into changes in lipid metabolism, which is helpful to tailoring AD personalized treatment strategies and revealing potential mechanism of disease and drug actions. The lipid changes in plasma indicated that the metabolic characteristics of adipocyte differentiation and suggested that DNP could affect adipocyte differentiation in AD patients with DNP treatment.

## Materials and Methods

### Subjects

This study was approved by the ethics committee of Shanghai Jiao Tong University Affiliated 60 People’s Hospital and complied with the Declaration of Helsinki. A total of 85 Chinese patients and healthy control were enrolled in this study. All individuals were screened using the Mini-Mental State Exam (MMSE). According to MMSE, patients were classified as having mild dementia (MMSE score: 21–26) and moderate dementia (MMSE score: 11–20). We categorized the participants into two groups; the first is a DNP responder group (DNP-R, *n* = 47), defined as AD patients with DNP treatment for at least 3 months, ΔMMSE (the difference between the first and the last MMSE score, which was acquired after at least 3 months of follow-up) ≥0. The second is a DNP nonresponder group (DNP-N, *n* = 38) was defined as ΔMMSE <0. Blood samples were taken in standard sealed sampling tubes with EDTA anticoagulation. The plasma was separated *via* centrifugation (3,000 rpm, 15 min), and samples were aliquoted and stored at −80°C until further processing.

### Metabolomics Analyses

Metabolomics analysis was performed on an Agilent 7890A gas chromatography system coupled to an Agilent 5975C inert MSD system (Agilent Technologies Inc., CA, USA). An HP-5 ms fused-silica capillary column (30 m × 0.25 mm × 0.25 μm; Agilent J&W Scientific, Folsom, CA, USA) was utilized to separate the derivatives. Helium (>99.999%) was used as a carrier gas at a constant flow rate of 1 ml/min through the column. For the extraction of metabolites, sample derivatization, GC-MS analysis, data preprocessing, and statistical analysis, please refer to the previous published protocols (Wan et al., 2018).

### Plasma Adiponectin Test

The concentrations of total APN in plasma were quantified using an ELISA system and 10 μl (100 dilution) plasma were assayed in duplicate by Human total Adiponectin/Acrp30 Quantikine Elisa Kits obtained for Techne Corporation Research and Diagnostics Systems Europe Limited.

### Genotyping of APOE and Adiponectin Genes

Peripheral blood was collected into EDTA Vacutainer tubes and stored at −80°C until analysis. The adiponectin-encoding gene (ADIPOQ) was genotyped using PCRLDR (ligase detection reaction) technologies. Genomic DNA was extracted from the peripheral blood samples using an AxyPrep-96 DNA blood kit (AXYGEN, USA). The APOE and ADIPOQ alleles were identified by allele-specific PCR technology. Genes rs429358 and rs7412 were analyzed for APOE gene polymorphisms, and ADIPOQ gene polymorphisms were analyzed by examining rs1501299 and rs2241766. In addition, subjects with at least one APOE ε4 allele were considered ε4 carriers, while those with ε2 or ε3 alleles were considered ε4 noncarriers. The sequences were analyzed using an ABI PRISM 3100 DNA Sequencer (Applied Biosystems, CA, USA).

### Statistics

Differences between genotypes were analyzed by the Kruskal–Wallis test, differences between two groups of continuous data were analyzed by using the nonparametric Mann–Whitney test, and the χ^2^ test and Fisher’s exact test were used to compare the rate of responders among the groups. Pearson correlation coefficients were used to compare samples and determine correlations. The diagnostic performance was assessed by the area under the receiver operator characteristic (ROC) curve. Cutoff values were calculated using sensitivity and specificity that maximized Youden’s index. Ninety-five percent confidence intervals were calculated for the area under the curve (AUC). Statistical significance was defined as *p* < 0.05.

## Results

### Demographic Characteristics and Routine Biochemical Profiling

Clinical and biochemical characteristics of responders and nonresponders to DNP among AD patients, AD patients without DNP treatment and nondemented controls were summarized in Table 1. The details of demographic characteristics and clinical features including acetylcholinesterase activities of the subjects were given in our previous study (Wan et al., 2018).

**Table 1:** Demographics and clinical features of the subjects.

	DNP responders	DNP non-responders
*N*	48	37
Age (year)	80.5 ± 7.6	81.2 ± 7.3
Gender, female (%)	18 (37.5%)	16 (43.2)
MMSE scores at baseline	21.0 ± 4.5	22.9 ± 5.2*a
MMSE scores	23.2 ± 4.5	**18.0 ± 5.3***a
at steady-state
MMSE scores change	2.26 (0–8)	−5.20 (−1 to −18***a)

a: Wilcoxon matched pairs test, DNP responders to DNP non-responders, *p < 0.05; ***p < 0.001.

### Association of Plasma APN Levels With the Response to DNP

**Figure 1:**
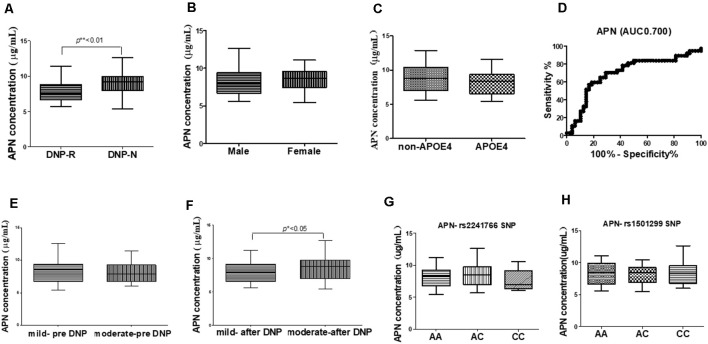
Comparison of plasma adiponectin (APN) levels among donepezil (DNP) treatment responder (DNP-R) and DNP treatment without responder (DNP-N; **A**), different sexes **(B)**, APOE ε4 **(C)**, different severity of AD before DNP treatment **(E)**, different severity of AD after DNP treatment **(F)**, ROC curves for models predicting incident DNP response with APN **(D)**, comparison of plasma APN levels and APN polymorphism of rs1501299 **(H)**, rs2241766 (**G**; Mann–Whitney test, *p < 0.05, **p < 0.01).

**Figure 2:**
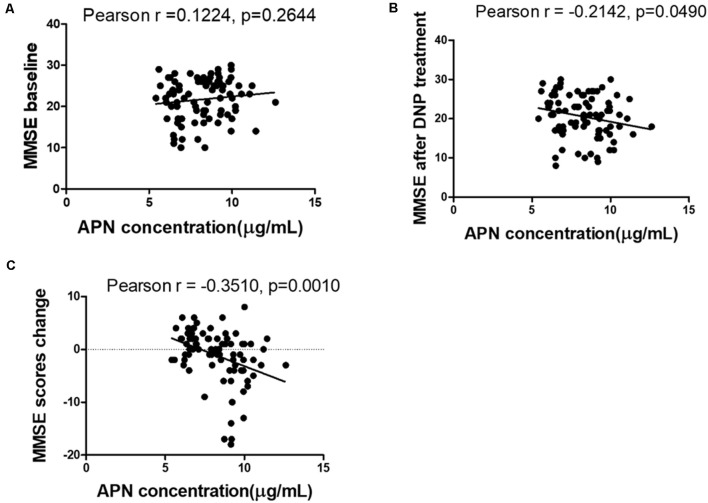
Correlation between APN levels and Mini-Mental State Examination (MMSE) scores among baseline **(A)**, after DNP treatment **(B)**, and change before and after treatment **(C)**.

The concentrations of plasma APN levels were 7.5 μg/ml and 9.2 μg/ml for DNP-R and DNP-N respectively (Figure 1A). Plasma APN levels were significantly decreased in DNP-R group compared to the AD and DNP-N group (7.5 μg/ml vs. 9.2 μg/ml, *p* < 0.05). There were no differences in plasma total APN between male and female subjects, APOE ε4 carriers and noncarriers and different severities of AD before DNP treatment (Figures 1B,C,E). Plasma APN levels were significantly decreased in the mild dementia group compared with the moderate dementia group after DNP treatment (Figure 1F). We performed a statistical analysis to evaluate the difference between the APN polymorphisms and response to DNP treatment. APN plasma concentrations with regards to the rs1015299 and rs2241766 genotypes are shown in Figures 1G,H. ADIPOQ polymorphisms were not related to plasma APN levels in our study. To assess the sensitivity and specificity of APN between groups of DNP-R and DNP-NR, we performed ROC analysis. As shown in Figure 1D, ROC curve analysis demonstrated that the AUC of APN calculated to be 0.7000 (95% confidence interval: 0.5801–0.8174, *p* = 0.002). As shown in Figures 2A–C, there was a significant negative correlation between plasma APN levels and MMSE scores after DNP treatment (*p* < 0.05) and MMSE score change (*p* < 0.01) in all DNP treatment subjects. Our results indicated that the six-month DNP treatment induced decrease of plasma APN levels in AD patients. It is necessary to determine whether the reduction in APN levels was related to the effects of DNP or the progression of the disease.

### Metabolite Profiling in DNP Response According to Adipogenic Differentiation and Biomarkers of DNP Response

**Figure 3:**
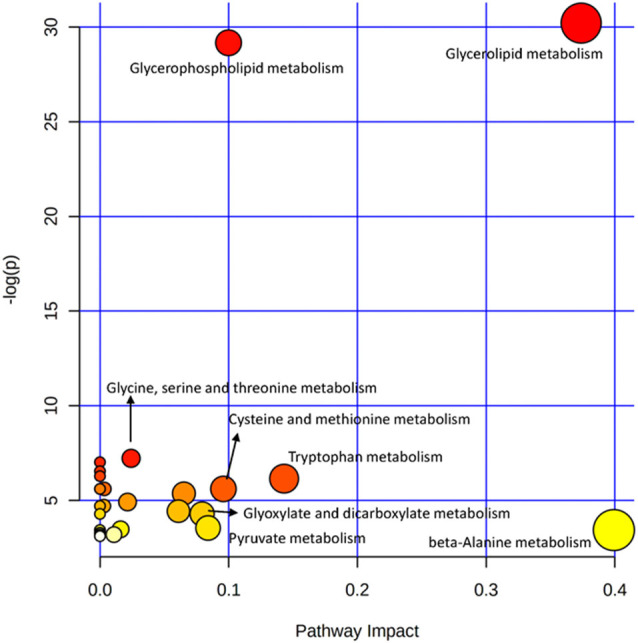
Plasma metabolic pathway analysis. All the matched pathways are displayed as circles. The color and size of each circle are based on the *p*-value and pathway impact value, respectively. The graph was obtained by plotting on the *y*-axis the log of *p*-values from the pathway enrichment analysis and on the *x*-axis the pathway impact values derived from the pathway topology analysis.

**Figure 4:**
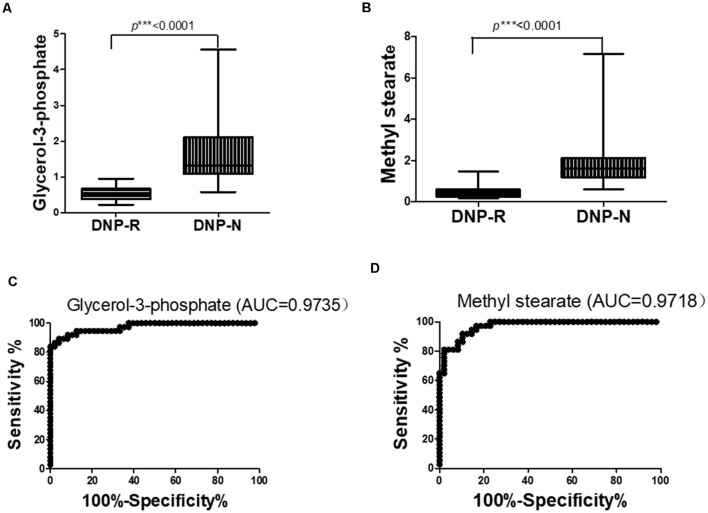
Changes in glycerol-3-phosphate **(A)** and methyl stearate **(B)** in plasma of DNP-R and DNP-N and ROC curves for models predicting incident DNP response with glycerol-3-phosphate **(C)** and methyl stearate (**D**; Unpaired, 2-tailed Student’s *t*-test. ***p < 0.001).

**Figure 5:**
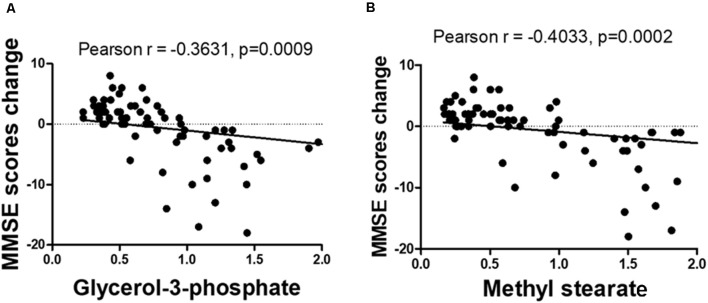
Correlation between glycerol-3-phosphate **(A)** and methyl stearate **(B)** levels and the change of before and after treatment of Mini-Mental State Examination (MMSE) scores.

The OPLS-DA analysis found significant differential metabolites among the two groups. Using a threshold of VIP >1.0 in the OPLS-DA models and applying a two-tailed Student’s *t*-test, 21 differential metabolites, of which 11 were decreased and 10 increased in DNP-R when comparing to DNP-N, were identified (Supplementary Table S1). Among the 28 metabolic pathways identified, four were significantly enriched (*p* < 0.05; impact >0.1). These findings demonstrated that four pathways, including glycerolipid metabolism, glycerophospholipid metabolism, tryptophan metabolism, and beta-alanine metabolism, were closely related to the DNP respond (Figure 3). Compared with DNP-N, DNP-R were characterized by lower levels of glycerol-3-phosphate and methyl stearate (Figures 4A,B). These differential metabolites are mainly involved in adipocyte differentiation. ROC curve analysis demonstrated that for glycerol-3-phosphate and methyl stearate, the AUC values were calculated to be 0.9735 (95% confidence interval: 0.9440–1.003, *p* = 0.001), and 0.9718 (95% confidence interval: 0.9447–0.9990, *p* = 0.001) respectively (Figures 4C,D). As shown in Figures 5A,B, there was a significant negative correlation between plasma glycerol-3-phosphate/methyl stearate levels and MMSE scores change (*p* < 0.001) in all DNP treatment subjects.

## Discussion

Lipids and adipokines research are an emerging area of biomedical interest, and may reflect pathology or even as the mediators of AD. Previous studies have suggested that DNP treatment has an influence not only on AChEIs, but also can increase serum APN levels (Pákáski et al., 2014). Inconsistently with previous self-controlled studies, in our cross-sectional study, plasma APN levels in DNP responders were lower than in DNP nonresponders. One reason for this discrepancy may be the difference of experimental designs. Our experimental design was cross-sectional and divided into two groups, the effective group and the ineffective group, according to the patients’ responses to DNP. The application of cross-sectional study with disease progression taken into account may contribute to the discrepancy.

In our study, we found that APN and small-molecule lipids can be used as potential biomarkers to evaluate the efficacy of DNP. The low concentration of APN in the DNP group suggests that DNP may reduce the level of APN in circulation. The results of metabolomics indicate that there was no change in the metabolic pathway of fatty acid metabolism and glucose metabolism in DNP effective group, suggesting that APN-related biological functions did not decrease in DNP effective group. Simultaneously, ADIPOQ polymorphisms were not related to plasma APN levels in our study, which suggested that subcutaneous or abdominal adipose tissue might not be the direct sources of APN. The result of our study suggests that we need to pay more attention to the sources and biological functions of increased APN in AD. One hypothesis about the lower APN levels in DNP responders is that DNP can decrease APN and be attributable to the treatment of AD. To confirm this hypothesis, the question of what would cause increased APN has to be discussed. First cytokines and transcription factors: extensive efforts have revealed various APN regulators, such as cytokines and transcription factors. Cytokines, such as tumor necrosis factor α, interleukin-1b (IL-1b), and interleukin-6 (IL-6), and IL-18, downregulate APN production (Liu and Liu, 2009). On the other hand, transcription factors such as peroxisome proliferator-activated receptor (PPARγ; Zhang et al., 1996), CCAAT-enhancer-binding protein α, and forkhead box O1 (FoxO1) upregulate APN expression (Kita et al., 2005). Second, elevated circulating APN levels was also found in chronic heart failure (Bozic et al., 2010; Celik and Yaman, 2010), caloric restriction (Cawthorn et al., 2014), and anorexia nervosa (Delporte et al., 2003). The hypothesis about high-level circulating APN was focused on local APN expression in bone marrow adipose tissue. That means that marrow adipose tissue was a source of circulating APN. We had some data about the healthy volunteers in Supplementary Figure S1: compared with the healthy control group, plasma APN levels were increased in AD patients newly diagnosed and without administration of DNP, but the difference was not statistically significant. Far less is known about why plasma APN increases in AD. The hyperadiponectinemia might be derived from an unexpected source: bone marrow adipose tissue. Adipogenesis is the process of cell differentiation from preadipocytes to adipocytes. The APN gene is generally used as adipocyte markers at the terminal stage of differentiation (Sarjeant and Stephens, 2012). The increased adipogenesis of both preadipocyte in MAT and fat tissue can result in elevated circulating APN.

Moreover, consistent with the change of APN levels, the metabolites that were associated with adipocyte differentiation and lipid synthesis, such as glycerol-3-phosphate and methyl stearate, were all decreased in DNP responder groups. The potential clinical implications of the present data include that: (1) DNP may work best in those with lower APN levels; and (2) DNP may decrease levels of APN. If DNP could decrease APN by the target on subcutaneous and visceral adipose tissue, DNP may have effects on cytokines and transcription factors that are involved in APN secretion. The previous findings showed that high levels of inflammatory cytokines observed in AD, and AChEIs treatment downregulates the release of these cytokines (Reale et al., 2005). From this point of view, DNP should be able to increase the secretion of APN from subcutaneous and visceral adipose tissue. We speculated that the target of DNP was MAT but not the subcutaneous and visceral adipose tissue.

Increased circulating APN can come from MAT. Although observations follow the well-documented increased bone marrow adipose tissue with age, it is unknown whether bone marrow adipose tissue can become dysfunctional, or whether specific types of bone marrow adipose tissue adipocytes are more likely to have deleterious effects in AD. APN might be one of the mediators of the fat-bone relationship, and a positive relationship exists between increased marrow fat and higher circulating levels of APN (Naot et al., 2017). Increased marrow adiposity has been shown to be an independent predictor of fracture, and higher marrow fat was associated with prevalent vertebral fracture (Schwartz et al., 2013). There are no reports about DNP’s effect on MAT, but there are some studies on the relationship of AChEIs to bone mass and hip fracture. DNP-treated mice had better bone quality than controls (Eimar et al., 2016). In elderly female patients with AD, the use of AChEIs might be associated with an enhanced fracture healing and minimized complications (Eimar et al., 2013). Elderly patients with AD who are at risk of developing osteoporosis may potentially benefit from therapy with DNP (Tamimi et al., 2012, 2017). The mechanism by which DNP enhances fracture healing is not clear. Since MAT is closely associated with bone, the effects of DNP on bone mass possibly rely on its effect on MAT. Marrow adipocytes arise from the mesenchymal stromal cell (MSC) in the bone marrow. As the progenitors of the bone-forming cells, MSC of patients with osteoporosis are reduced in proliferation, migration, and differentiation rates. Therefore, it is possible that DNP would also have suppressive effects on adipogenic differentiation of MSC and can decrease circulating APN levels.

Methyl stearate, which was used for characterizing adipogenic differentiation of human bone marrow-derived MSC (Lee et al., 2014) was significantly decreased in DNP-R compared to DNP-N. PPARγ is a transcription factor induced during adipocyte development of preadipocytes and remains abundantly expressed in differentiated adipocytes. The main effect of PPARγ activation on glucose metabolism is the increased generation of glycerol-3-phosphate for triglyceride synthesis (Nakamura et al., 2014). Glycerol-3-phosphate was significantly decreased in DNP-R group compared to DNP-N. PPARγ targets phosphoenolpyruvate carboxykinase (PEPCK), glucokinase (GK) and 6-phosphofurcto-2-kinase/fructose-2,6-bisphosphatase 3 (PFKFB3). Activated PPARγ induces PEPCK, GK, and PFKFB3. PEPCK induction switches the metabolic fate of pyruvate from oxidation to glycerol-3-phosphate synthesis. PEPCK and GK induction has resulted in activation of glucose metabolism and increased generation of glycerol-3-phosphate. That is evidence to support our hypothesis that DNP would have a suppressive effect on PPARγ and suppressed adipogenic differentiation.

Based on the results of lower APN concentrations of the DNP responders group in this study, the extended question is whether lower concentrations of APN can also reduce its physiological protection effects such as enhancing glucose utilization and fatty-acid combustion, which cause related adverse reactions. Metabolomics may provide metabolite-based biological system profiles and metabolic pathways. We did not find the difference between the DNP responders and nonresponders in pathways related to fatty acid biosynthesis and glycolysis/gluconeogenesis. If the increased APN levels circulating in AD come from marrow adipocytes, the question of whether APN from bone marrow has the same biological function as APN from adipose tissues remains unanswered.

There are several limitations to the present study. The small sample size in our study limits the interpretation of the findings and due to the cross-sectional design, we have no data regarding baseline levels of APN and lipids prior to the use of DNP. We did not record body mass index, education levels, and concomitant diseases, all of which could have affected the levels of APN. We used only the MMSE test to measure cognitive impairment and cognitive changes and to document an individual’s response to treatment. We did not record education levels, which could have affected the score at the MMSE cutoff. The pilot study findings that DNP responders had a lower level than nonresponders and may relate to bone marrow remains to be verified by additional studies.

In summary, in the present study, we have observed striking differences in the plasma levels of APN, glycerol-3-phosphate, and methyl stearate in DNP-R compared with DNP-NR, which are likely to have important implications in the mechanism of drug action. Furthermore, the concentration of APN, glycerol-3-phosphate, and methyl stearate might potentially be a sensitive DNP responder biomarker. Further studies are needed to confirm the hypothesis that DNP has suppressive effect on adipogenesis of bone marrow MSC.

## Data Availability Statement

The raw data supporting the conclusions of this article will be made available by the authors, without undue reservation, to any qualified researcher.

## Ethics Statement

The studies involving human participants were reviewed and approved by Ethics committee of Shanghai Jiao Tong University Affiliated Sixth People’s Hospital. The patients/participants provided their written informed consent to participate in this study.

## Author Contributions

LW was responsible for the study conception and design, interpretation of the analysis, and writing of the manuscript. JL participated in the collection of data for clinical research and was responsible for the acquisition of data. JH was responsible for the acquisition of data and critical revision of the manuscript. YH was responsible for the acquisition of data and critical revision of the manuscript. SJ had revised and polished the English writing of the manuscript. CG designed the study and helped draft the manuscript. All authors contributed to the article and approved the submitted version.

## Conflict of Interest

The authors declare that the research was conducted in the absence of any commercial or financial relationships that could be construed as a potential conflict of interest.

## Supplementary Material

FIGURE S1 Comparison of plasma adiponectin, glycerol-3-phosphate, and methyl stearate levels in four groups: healthy controls (control, *n* = 30) that were matched to AD cases for gender and age and MMSE >27; Alzheimer’s disease patients (AD, *n* = 30) were newly diagnosed for sporadic AD according to the criteria of MMSE and without administration of DNP; AD with DNP treatment for at least 3 months, ΔMMSE (the difference between the first and the last MMSE score, which was acquired after at least 3 months of follow-up) ≥0 would be defined as DNP responder (DNP-R, *n* = 47; one-way ANOVA, **p* < 0.05, ***p* < 0.01, ****p* < 0.001).

TABLE S1 Metabolic differences among DNP treatment responder (DNP-R) and DNP treatment non-responder (DNP-N).
