# Supplementary figures and images for: Association Between Peripheral Adiponectin and Lipids Levels and the Therapeutic Response to Donepezil Treatment in Han Chinese Patients With Alzheimer’s Disease
Source: Front Aging Neurosci. 2020 Sep 11;12:532386. doi: 10.3389/fnagi.2020.532386 (PMC7518373; doi:10.3389/fnagi.2020.532386)

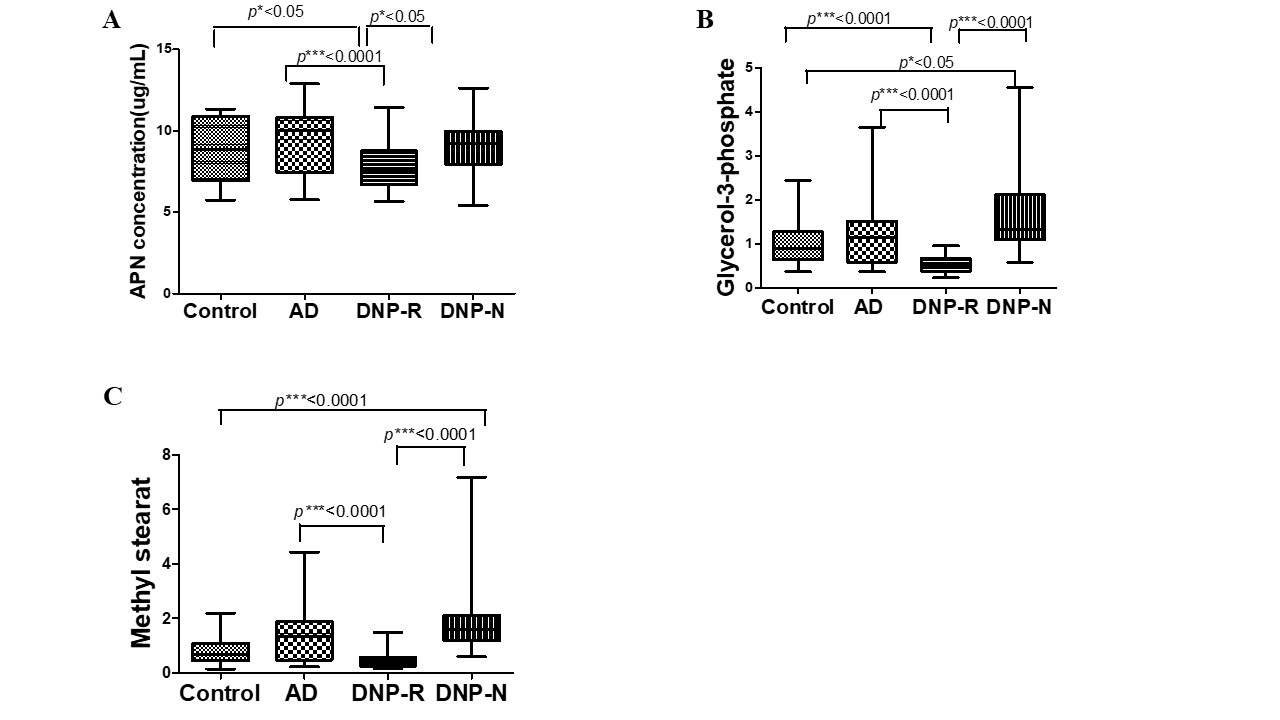

Supplement: FIGURE S1 — Comparison of plasma adiponectin, glycerol-3-phosphate, and methyl stearate levels in four groups: healthy controls (control, n = 30) that were matched to AD cases for gender and age and MMSE >27; Alzheimer’s disease patients (AD, n = 30) were newly diagnosed for sporadic AD according to the criteria of MMSE and without administration of DNP; AD with DNP treatment for at least 3 months, ΔMMSE (the difference between the first and the last MMSE score, which was acquired after at least 3 months of follow-up) ≥0 would be defined as DNP responder (DNP-R, n = 47; one-way ANOVA, *p < 0.05, **p < 0.01, ***p < 0.001). [file Image_1.TIF]
