# Supplementary material for: Association Between Peripheral Adiponectin and Lipids Levels and the Therapeutic Response to Donepezil Treatment in Han Chinese Patients With Alzheimer’s Disease
Source: Front Aging Neurosci. 2020 Sep 11;12:532386. doi: 10.3389/fnagi.2020.532386 (PMC7518373; doi:10.3389/fnagi.2020.532386)
Supplement: TABLE S1 — Metabolic differences among DNP treatment responder (DNP-R) and DNP treatment non-responder (DNP-N). [file Table_1.docx]

Table S1 Metabolic differences among DNP treatment responder (DNP-R) and DNP treatment nonresponder (DNP-N).

| No. | Metabolites | RT (min) | M/Z | VIP | p-value | FC |
| --- | --- | --- | --- | --- | --- | --- |
| 1 | methyl stearate | 24.81 | 87 | 4.31 | 1.38E-11 | -2.02 |
| 2 | 1-oleoylglycerol | 28.64 | 129 | 2.71 | 1.07E-04 | -0.52 |
| 3 | glycerol-3-phosphate | 20.77 | 357 | 4.43 | 2.20E-12 | -1.61 |
| 4 | glycerol-2-phosphate | 20.28 | 243 | 4.03 | 6.07E-10 | -0.34 |
| 5 | glycerol | 11.71 | 205 | 2.35 | 8.94E-04 | -0.36 |
| 6 | 2-keto-l-gluconic acid | 20.98 | 292 | 2.35 | 8.97E-04 | -0.54 |
| 7 | threonic acid | 17.69 | 292 | 1.49 | 3.84E-02 | -0.41 |
| 8 | glyceric acid | 12.91 | 189 | 1.77 | 1.38E-02 | -0.29 |
| 9 | dihydrocholesterol | 31.46 | 215 | 2.61 | 1.97E-04 | -0.35 |
| 10 | campesterol | 32.3 | 343 | 2.27 | 1.36E-03 | -0.40 |
| 11 | beta-sitosterol | 33.22 | 129 | 2.03 | 4.48E-03 | -0.23 |
| 12 | 3-methyl-2-ketobutyric acid | 8.66 | 202 | 1.48 | 4.10E-02 | 0.14 |
| 13 | DHA | 27.73 | 117 | 1.45 | 4.44E-02 | 0.15 |
| 14 | lactic acid | 7.19 | 117 | 1.57 | 2.89E-02 | 0.23 |
| 15 | myo-inositol-1-phosphate | 26.88 | 318 | 1.56 | 3.10E-02 | 0.25 |
| 16 | lysine | 22.69 | 174 | 1.50 | 3.73E-02 | 0.29 |
| 17 | tryptophan | 25.5 | 202 | 2.18 | 2.13E-03 | 0.29 |
| 18 | cysteine | 17.58 | 220 | 2.07 | 3.67E-03 | 0.35 |
| 19 | beta-alanine | 14.98 | 174 | 1.54 | 3.23E-02 | 0.36 |
| 20 | 4-hydroxyproline | 17.02 | 230 | 1.80 | 1.19E-02 | 0.47 |
| 21 | homoserine | 15.21 | 218 | 2.25 | 1.47E-03 | 0.96 |

RT: Retention times in minutes； M/Z: mass to charge ratio; VIP:VIP scores obtained from multivariate statistical OPLS-DA model; *p*-value: Student’s t test，threshold value was 0.05; FC: Fold change between DNP-R to DNP-N, where the positive value means that the average mass response ofDNP-R is higher than DNP-N
